# Thaliporphine Derivative Improves Acute Lung Injury after Traumatic Brain Injury

**DOI:** 10.1155/2015/729831

**Published:** 2015-02-01

**Authors:** Gunng-Shinng Chen, Kuo-Feng Huang, Chien-Chu Huang, Jia-Yi Wang

**Affiliations:** ^1^Graduate Institute of Medical Sciences, College of Medicine, Taipei Medical University, Taipei 110, Taiwan; ^2^Division of Orthodontics & Dentofacial Orthopedics and Pedodontics, Department of Dentistry, Tri-Service General Hospital, Taipei 114, Taiwan; ^3^Division of Neurosurgery, Department of Surgery, Taipei Tzu Chi Hospital, Buddhist Tzu Chi Medical Foundation, New Taipei City 23142, Taiwan; ^4^School of Medicine, Tzu Chi University, Hualien 97004, Taiwan; ^5^Teaching Department of Medicine, China Medical University, Taichung 40402, Taiwan; ^6^Department of Physiology, School of Medicine, College of Medicine, Taipei Medical University, Taipei 110, Taiwan

## Abstract

Acute lung injury (ALI) occurs frequently in patients with severe traumatic brain injury (TBI) and is associated with a poor clinical outcome. Aquaporins (AQPs), particularly AQP1 and AQP4, maintain water balances between the epithelial and microvascular domains of the lung. Since pulmonary edema (PE) usually occurs in the TBI-induced ALI patients, we investigated the effects of a thaliporphine derivative, TM-1, on the expression of AQPs and histological outcomes in the lung following TBI in rats. TM-1 administered (10 mg/kg, intraperitoneal injection) at 3 or 4 h after TBI significantly reduced the elevated mRNA expression and protein levels of AQP1 and AQP4 and diminished the wet/dry weight ratio, which reflects PE, in the lung at 8 and 24 h after TBI. Postinjury TM-1 administration also improved histopathological changes at 8 and 24 h after TBI. PE was accompanied with tissue pathological changes because a positive correlation between the lung injury score and the wet/dry weight ratio in the same animal was observed. Postinjury administration of TM-1 improved ALI and reduced PE at 8 and 24 h following TBI. The pulmonary-protective effect of TM-1 may be attributed to, at least in part, downregulation of AQP1 and AQP4 expression after TBI.

## 1. Introduction

Acute lung injury (ALI) commonly occurs after traumatic brain injury (TBI) [1, 2]. Respiratory failure is the most common complication of the nonneurological organ system in patients with TBI [3]. ALI has been reported for a long time, but it remains poorly understood because of the complexity of its pathophysiological mechanisms [4]. ALI is characterized by marked pulmonary vascular extravasation and intra-alveolar accumulation of protein-rich edema fluid with perivascular edema [5–7]. Water homeostasis is a crucial element of major pathophysiological processes occurring in the respiratory system. Aquaporins (AQPs), water channel proteins, play a pivotal role in this homeostasis [8–10]. Modulation of lung AQPs expression and function by using pharmacological or genetic methods may have clinical utility [11, 12]. AQPs including AQP1 and AQP4 regulate water movement across the epithelial and endothelial barriers of the lung [4, 13]. Specific deletions of AQPs in mice markedly decreased water movement across air space-capillary barriers [14]. AQP1 is expressed in pleural membranes, when pleural fluid accumulation was observed in the peritoneal cavity [14]. AQPs are also involved in lung growth and angiogenesis [15]. However, the roles of AQP1 and AQP4 expression in pulmonary edema remain controversial. Previous studies have shown that* AQP1* knockout inhibits water transport [16] and reduces sublaryngeal edema [13]. Downregulation of AQP1 expression in alveolar microvessels may act as a compensatory mechanism to protect against the formation of excessive pulmonary edema (PE) in chronic heart failure [17]. However, there have also been reports suggesting that pulmonary inflammation and edema were associated with a marked reduction in the* AQP1* mRNA expression level in the lung [18, 19] and the* AQP1* mRNA expression level was negatively correlated with the level of the wet-to-dry weight ratio (wet/dry weight ratio) [20].* AQP4* mRNA expression is upregulated in alveolar type II (ATII) cells [21] and AQP4 is believed to regulate fluid exchange between the alveolar space and the alveolar epithelium barrier in ALI [22]. However,* AQP4* deletion did not impair water transport in the airways [23]. Thaliporphine is a phenolic aporphine alkaloid obtained from Chinese herbs that possesses antioxidant and *α*-1 adrenoceptor antagonistic activities. A previous study showed that thaliporphine reduces the risk of cardiac muscle necrosis and arrhythmia after heart ischemia [24]. Moreover, the positive effects of thaliporphine may be attributed to the suppression of TNF-*α*, NO·, and O_2_·^−^ production [25]. Hence, this study was aimed at investigating the effect of TM-1 on TBI-induced ALI and AQPs expression in the lung.

## 2. Materials and Methods

### 2.1. Drug Preparation

TM-1 is a derivative of (+)-thaliporphine dissolved in 100% dimethyl sulfoxide (DMSO) (30 mg in 1 mL DMSO). A dose of 10 mg/kg was used for intraperitoneal injection (i.p.).

### 2.2. Surgical Procedures

All animals (male Sprague-Dawley rats; body weight 250–300 g) were treated in accordance with the International Guidelines for animal research. The study design was approved by the animal ethics committee (Approval number LAC-100-0143) of Taipei Medical University. The animals were housed in groups in a controlled environment of temperature (21–25°C) and humidity (45–50%) with a 12-h light/dark cycle and* ad libitum *access to pellet chow and water. The CCI injury procedure was performed as described previously [26]. An impact velocity of 4 m/s and a deformation depth of 2 mm below the dura were used. The bone flap was immediately replaced and sealed, and the scalp was closed with sutures. Body temperature was monitored throughout surgery by using a rectal probe; the temperature was maintained at 37.0 ± 0.5°C using a heated pad. The rats were placed in a heated cage to maintain body temperature while recovering from anesthesia. Sham-operated rats were subjected to craniotomy as described previously but not to CCI; the impact tip was placed lightly on the dura before sealing the wound. After TBI or sham surgery, all animals were housed under the aforementioned conditions.

### 2.3. Time Point for TM-1 Administration

The rats received an IP TM-1 injection of 10 mg/kg (0.1 cc) or vehicle injection (0.1 cc DMSO) 3 or 4 h after TBI. The animals were sacrificed 8 or 24 h after TBI. The TM-1 administration dose was selected on the basis of pilot studies conducted in our laboratory, in which doses of 5 and 10 mg/kg were tested; the 10-mg/kg dose showed neuroprotective effects by improving behavioral deficits (unpublished results). After the CCI injury procedure, the rat tissues were processed for histological examination, wet/dry weight ratio calculation, real-time quantitative reverse transcription (RT)-PCR, western blotting, and lung injury scoring at 8 or 24 h (*n* = 5/time point). Five sham-operated rats were used for analysis and were administered a corresponding volume of the vehicle.

### 2.4. PE Was Evaluated by the Wet/Dry Weight Ratio

The wet/dry weight ratio was used as an index of PE after TBI. To measure the index, the fresh upper right lobe of the lung was harvested (*n* = 5). Tissue weight was measured immediately after its excision (wet weight), and the tissue was then dried in an oven at 60°C for 5 days to attain a constant weight. The wet/dry weight ratio was calculated by dividing the wet weight by the dry weight as described previously [27].

### 2.5. RNA Extraction, Reverse Transcription, and Real-Time Quantitative PCR (qPCR)

Right lung tissues from the rats with TBI or sham-operated rats (*n* = 5) were removed after postanesthesia decapitation. Total RNA was extracted from half of the right middle lobe of the lung by using TRIzol RNA isolation reagents (Life Technologies, USA). The purity and quality of total extracted RNA were confirmed by determining the ratio of absorbance at 260 nm to that at 280 nm. Total RNA was subjected to reverse transcription by using the ReverTra Ace Set Kit according to the manufacturer's protocol (Purigo, Taiwan). The RT mixtures were diluted and used as templates in subsequent qPCR or stored at −20°C. qPCR analysis was performed using a Rotor-Gene Q detector (Qiagen, USA). For mRNA measurement, diluted cDNA was amplified using the QuantiFast SYBR Green PCR kit. Using the PCR kit, thermal cycling was initiated with a first denaturation step of 5 min at 95°C, followed by 40 cycles of 95°C for 10 s and 60°C for 30 s. Primers used for the qPCR assay were as follows: 5′-GACACCTCCTGGCTATTGACTACA-3′ (*forward*) and 5′-CCGCGGAGCCAAAGG-3′ (*reverse*) for rat* AQP1*, 5′-AGCCTGGGATCCACCATC-3′ (*forward*) and 5′-TGCAATGCTGAGTCCAAAGC-3′ (*reverse*) for rat* AQP4*, 5′-GACCCAGATCATGTTTGAGACCTTC-3′ (*forward*) and 5′-GAGTCCATCACAATGCCWGTGG-3′ (*reverse*) for rat *β-actin*, and 5′-GACATGCCGCCTGGAGAAAC-3′ (*forward*) and 5′-AGCCCAGGATGCCCTTTAGT-3′ (*reverse*) for rat* Gapdh*. The Rotor-Gene Q detector synchronized with the thermal cycler measured fluorescent emission during each extension step. Each sample was run in triplicate. Relative standard curves for the candidate gene and the rat *β-actin* and* Gapdh* gene were plotted each time the genes were analyzed. The comparative threshold cycle (Ct) value of the *β-actin *and* Gapdh* genes, which were constant irrespective of experimental conditions, was used as reference genes. All PCR products were analyzed in the geometric range of the exponential phase during PCR amplification.* AQP1*,* AQP4*, *β-actin*, and* Gapdh* mRNA levels were calculated by the Ct method [28]. The reference genes were individually *β-actin* and* Gapdh* for mRNA. The resulting change in ΔCt values (expressed as ΔΔCt) was converted to a linear form using 2^(−ΔΔCt)^. This linear value was used in subsequent statistical analyses.

### 2.6. Western Blotting

Total proteins were extracted from tissues from half of the non-fixed right middle lobe of the lung of the rats with TBI or sham-operated rats by using RIPA buffer (25 mM Tris-HCl, pH 7.6, 150 mM NaCl, 1% NP-40, 1% sodium deoxycholate, 0.1% SDS) containing protease inhibitor cocktail (complete, Mini, EDTA-free; Roche Applied Science, Germany) and homogenized using a BBX24 Bullet Blender homogenizer (Next Advance, Inc., USA). After quantitative analysis with the Bradford protein assay by using Bio-Rad Dye Reagent (500-0006, Bio-Rad, USA), the protein samples were denatured with 5x sample buffer (10% SDS, 25% beta-mercaptoethanol, 50% glycerol, 0.25 M Tris-HCl, pH 6.8, 0.01% bromophenol blue) and incubated in a water bath at 95°C for 10 min. The amounts of tissue protein lysates loaded into 10% SDS-polyacrylamide gels and resolved by standard electrophoresis (Novex, Carlsbad, CA, USA) were 30 *μ*g for AQP1 and 100 *μ*g for AQP4, respectively. The gels were transferred onto PVDF filters incubated with a specific primary antibody to AQP1 (1 : 1000) (Santa Cruz Biotechnology, Inc., USA) and AQP4 (1 : 1000) (Santa Cruz Biotechnology, Inc., USA). HRP conjugated secondary antibodies were goat anti-mouse IgG (1 : 1000) for *β*-actin and goat anti-rabbit IgG (1 : 1000) for AQP1 and AQP4. These secondary antibodies were purchased from the Jackson ImmunoRes (West Grove, PA). All blots were reported with beta-actin as an internal standard (1 : 5000) (Millipore, USA). Bands of interest were visualized using ECL reagents (PerkinElmer, Waltham, MA, USA) and quantified using the UVP BioImaging system (Biospectrum AC Imaging System, CA, USA) and ImageJ software (National Institutes of Health, USA) [28].

### 2.7. Analysis of Lung Injury

Fresh right lower lung lobes were immediately fixed in 10% formalin and processed using the paraffin-embedding technique. Slides were stained with hematoxylin and eosin. The lung injury score was analyzed using a semiquantitative scoring system blinded to the treatment group. The lung injury scoring system included the score of neutrophils in the alveolar space, neutrophils in the interstitial space, hyaline membrane formation, proteinaceous debris filling the air spaces, and alveolar septal thickening. These criteria were accessible to both laboratory researchers and pathologists [29]. Ten microscopic fields from each slide were analyzed. The sums of tissue slides were averaged to evaluate the severity of lung injury.

### 2.8. Statistical Analyses

All data are presented as mean ± SEM. Between-group comparisons were made by one-way analysis of variance with the nonparametric Kruskal-Wallis test with Dunn's multiple comparison posttest. Instat 3 software (GraphPad Software Inc.; San Diego, CA, USA) was used for all analyses. *P* values of <0.05 were considered statistically significant. Pearson's regression analysis was conducted to analyze the correlation between the wet/dry weight ratio and the lung injury score in SAS version 7.1.

## 3. Results

### 3.1. TM-1 Administration 3 or 4 h after Injury Reduced Pulmonary Edema Developed at 8 or 24 h after TBI

**Figure 1:**
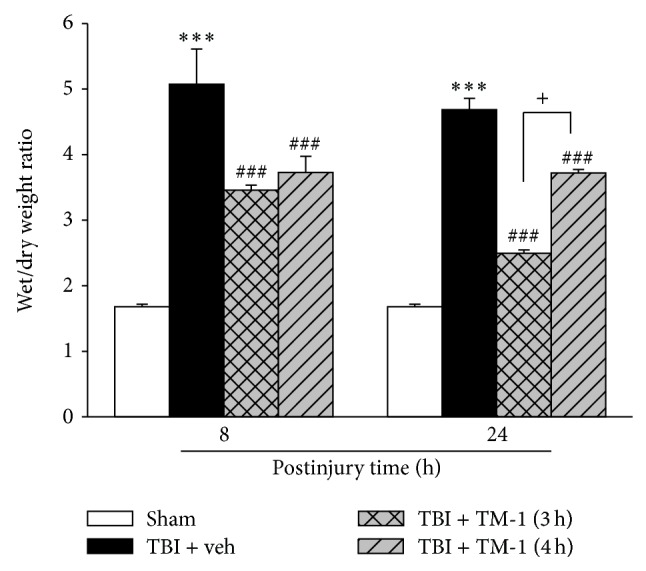
TM-1 administered 3 or 4 h after injury reduced the TBI-induced lung wet/dry weight ratio measured 8 and 24 h after TBI. Decrease in the wet/dry weight ratio at 3 or 4 h after injury with TM-1 administration (10 mg/kg) when the rats were sacrificed at 8 or 24 h after injury, respectively. Data are expressed as mean ± SD (*n* = 5/group). ^***^ *P* < 0.001 TBI + veh group compared with the sham-operated group; ^###^ *P* < 0.001 TM-1 treated groups compared with the TBI + veh groups; ^+^ *P* < 0.05, comparison between wet/dry weight ratios of rats sacrificed at 24 h following TM-1 administration (10 mg/kg) at 3 and 4 h after injury.

The wet/dry weight ratio, which reflects edema formation, of lung samples harvested from the rats with TBI dramatically increased at 8 and 24 h after injury compared with that of lung samples harvested from the sham-operated rats. Following TM-1 administration (10 mg/kg, i.p.) at 3 or 4 h after injury, the lung wet/dry weight ratio significantly decreased at 8 h or 24 h after injury in the TM-1 treated group compared with the TBI + veh group. Significant difference was observed between the rats administered TM-1 (10 mg/kg) 3 or 4 h after injury and sacrificed 24 h after injury (Figure 1).

### 3.2. TM-1 Administration at 3 or 4 h after Injury Diminished the mRNA Expression of* AQP1* in Pulmonary Injury at 8 or 24 h after TBI

**Figure 2:**
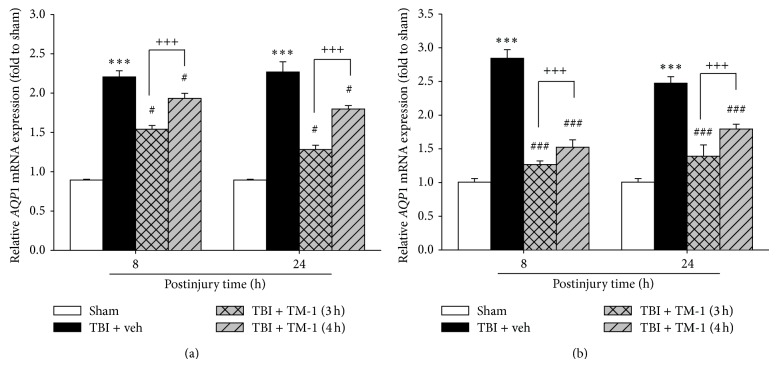
TM-1 administered 3 or 4 h after injury reduced the TBI-induced AQP1 mRNA expression measured 8 and 24 h after TBI. Relative* AQP1* mRNA expression was measured by reverse transcription Q-PCR using *β-actin* (a) and* Gapdh* (b) as reference genes.* AQP1* mRNA expression significantly decreased following TM-1 administration (10 mg/kg) at 3 and 4 h after injury in the rats sacrificed 8 or 24 h after injury, respectively. Compared with the TBI + veh groups, the TM-1 treated groups exhibited significantly decreased mRNA expression. Data are expressed as mean ± SD (*n* = 5/group). ^***^ *P* < 0.001 TBI + veh group compared with the sham-operated group; ^#^ *P* < 0.05 and ^###^ *P* < 0.0001 TM-1 treated group compared with the TBI + veh groups; ^+++^ *P* < 0.001, comparison between mRNA expression following TM-1 administration (10 mg/kg) 3 and 4 h after injury in the rats sacrificed 8 or 24 h after TBI.

Eight and 24 h after injury, the mRNA expression of* AQP1 *notably increased in the TBI + veh group (TBI treated with vehicle) compared with the sham-operated group. This increase in the mRNA expression was significantly attenuated by TM-1 administration at 3 or 4 h after injury and sacrifice at 8 or 24 h, respectively. Significant difference was observed between the rats administered TM-1 (10 mg/kg) at 3 or 4 h after injury. Similar measured patterns in* AQP1* mRNA expression were observed using the two reference genes, *β-actin* and* Gapdh* (Figures 2(a) and 2(b)).

### 3.3. TM-1 Administration 3 or 4 h after Injury Diminished the Protein Expression of AQP1 in Pulmonary Injury 8 or 24 h after TBI

**Figure 3:**
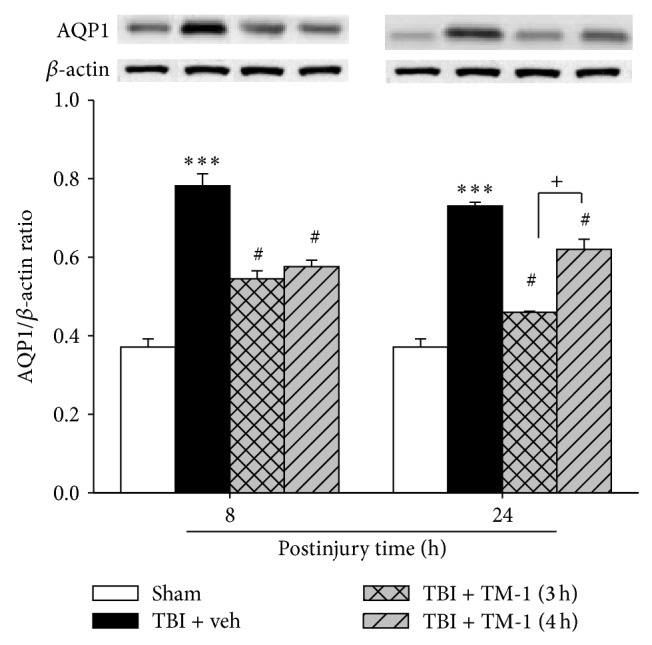
TM-1 administered 3 or 4 h after injury reduced TBI-induced lung AQP1 protein expression levels measured 8 and 24 h after TBI. The AQP1 protein level showed a marked decrease following TM-1 administration 3 or 4 h after injury (10 mg/kg), respectively. Rats were sacrificed 8 or 24 h after TBI. Data are expressed as mean ± SD (*n* = 5/group). ^***^ *P* < 0.001 compared with the sham-operated group; ^#^ *P* < 0.05 compared with the untreated groups; ^+++^ *P* < 0.001, comparison between mRNA expression following TM-1 administration (10 mg/kg) 3 and 4 h after injury in the rats sacrificed 8 and 24 h after TBI; ^+^ *P* < 0.05, comparison between protein levels following TM-1 administration (10 mg/kg) 3 and 4 h after injury in the rats sacrificed 24 h after TBI.

Eight and 24 h after injury, the protein level expression of AQP1 notably increased in the TBI + veh group compared with the sham-operated group (Figure 3). A significant reduction in AQP1 protein expression was attenuated by TM-1 administration at 3 or 4 h after TBI and AQP1 protein levels examined when animals were sacrificed at 8 h or 24 h after TBI. There was no significant difference in the TM-1 administration group whether treatment was given at 3 or 4 h post injury for animals subsequently sacrificed at either 8 or 24 h after TBI.

### 3.4. TM-1 Administration at 3 or 4 h after Injury Diminished the mRNA Expression of* AQP4* in Pulmonary Injury at 8 and 24 h after TBI with *β-actin* and* Gapdh *Reference Genes

**Figure 4:**
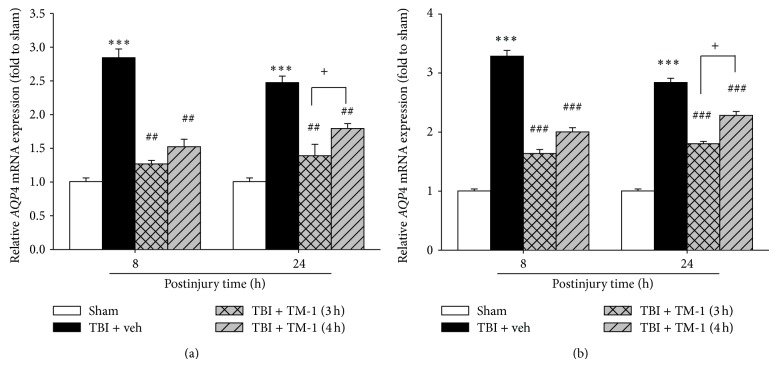
TM-1 administered 3 or 4 h after injury reduced the TBI-induced* AQP4* mRNA expression measured 8 and 24 h after TBI. (a) *β*-*actin* and (b)* Gapdh* as reference genes.* AQP4* mRNA expression significantly decreased following TM-1 administration (10 mg/kg) 3 and 4 h after injury in the rats sacrificed 8 or 24 h after injury, respectively. Compared with the TBI + veh groups, the TM-1 treated groups exhibited significantly decreased mRNA expression. Data are expressed as mean ± SD (*n* = 5/group). ^***^ *P* < 0.001 TBI + veh group compared with the sham-operated group; ^##^ *P* < 0.01 and ^###^ *P* < 0.001 compared with the TBI + veh groups; ^+^ *P* < 0.05, comparison between mRNA expression following TM-1 administration (10 mg/kg) 3 and 4 h after injury in the rats sacrificed 8 or 24 h after TBI.

Three and 4 h after injury, TM-1 administration substantially reduced* AQP4 *mRNA expression in the treated group 8 and 24 h after injury compared with the TBI + veh group (Figures 4(a) and 4(b)). Significant difference was observed between the rats administered TM-1 (10 mg/kg) at 3 or 4 h after injury and sacrificed at 24 h after injury (Figures 4(a) and 4(b)).

### 3.5. TM-1 Administration at 3 or 4 h after Injury Diminished the mRNA and Protein Expression of AQP4 in Pulmonary Injury 8 and 24 h after TBI

**Figure 5:**
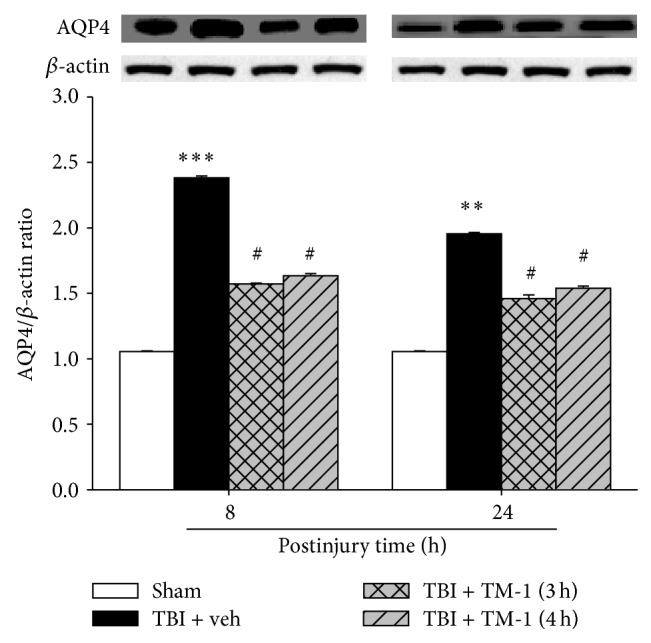
TM-1 administered 3 or 4 h after injury reduced the TBI-induced AQP4 protein level measured 8 and 24 h after TBI. The AQP4 protein level showed a marked decrease following TM-1 administration (10 mg/kg) 3 and 4 h after injury, respectively. Rats were sacrificed 8 or 24 h after TBI. Data are expressed as mean ± SD (*n* = 5/group). ^**^ *P* < 0.01 and ^***^ *P* < 0.001 TBI + veh group compared with the sham-operated group; ^#^ *P* < 0.05 TM-1 treated groups compared with the TBI + veh groups; no significance in AQP4 protein expression following TM-1 administration (10 mg/kg) at 3 and 4 h after injury in the rats sacrificed at 8 or 24 h after TBI.

Three and 4 h after injury, TM-1 administration substantially reduced AQP4 protein level expression in the TM-1 treated group 8 and 24 h after injury compared with the TBI + veh group. Significant difference was observed between the rats administered TM-1 (10 mg/kg) 3 or 4 h after injury and sacrificed 24 h after injury (Figure 5).

### 3.6. TM-1 Administration 3 or 4 h after Injury Improved Pathological Changes 8 and 24 h after TBI

**Figure 6:**
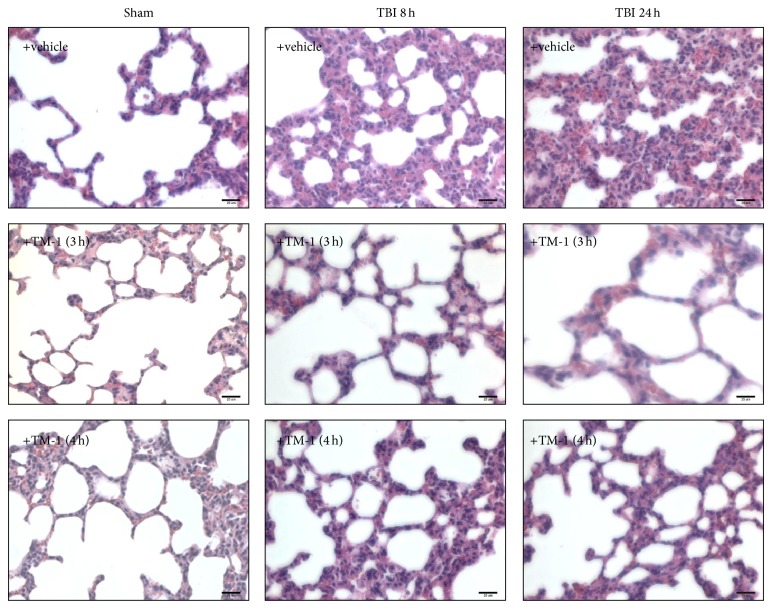
TM-1 administered 3 or 4 h after injury reduced alveolar swelling with infiltration of inflammatory cells 8 and 24 h after TBI by H&E staining. The vehicle-treated sham-operated group showed a normal alveolar morphology. Alveolar swelling and infiltration of inflammatory cells increased 8 and 24 h in vehicle-treated TBI rats. Alveolar swelling and infiltration of inflammatory cells decreased following TM-1 administration 3 and 4 h after injury. Rats were sacrificed 8 or 24 h after TBI. Bar = 25 *μ*m.

Sham-operated rats (vehicle-treated group) showed a normal alveolar morphology (Figure 6). Eight and 24 h after TBI, infiltration of numerous neutrophils in interstitial spaces, hemorrhage, and marked swelling of alveolar walls were observed in the rats with ALI. Extensive interstitial edema and infiltration of inflammatory cells were the same as these which have been previously described [30]. These pathological changes were attenuated in the rats administered TM-1 (10 mg/kg) at 3 or 4 h after injury and sacrificed at 8 or 24 h after TBI (Figure 6, middle and lower panels). No significant pathological changes were observed in the vehicle-treated sham-operated group.

### 3.7. TM-1 Administration 3 or 4 h after Injury Improved the Lung Injury Score 8 and 24 h after TBI

**Figure 7:**
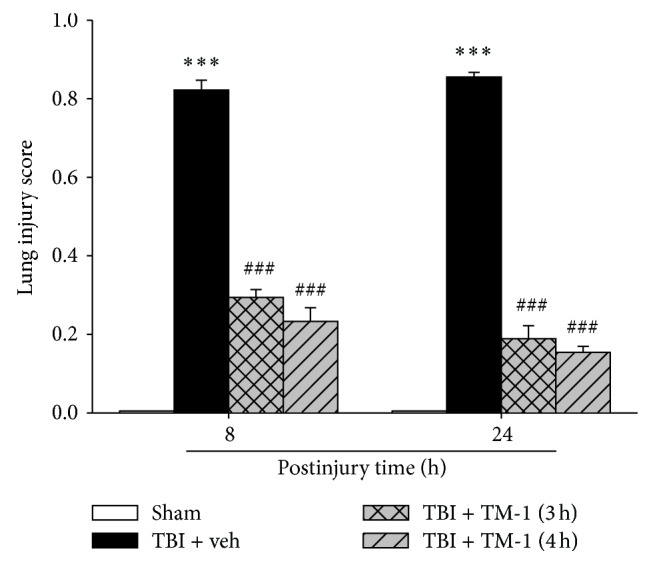
TM-1 administered 3 or 4 h after injury reduced the TBI-induced lung injury score 8 and 24 h after TBI. Significant difference was observed between the sham-operated and TBI groups. The lung injury score decreased with TM-1 administration (10 mg/kg) 3 and 4 h after injury. Rats were sacrificed 24 h after TBI. The lung injury score decreased following TM-1 administration (10 mg/kg) 3 and 4 h after injury. Rats were sacrificed 24 h after TBI. Data are expressed as mean ± SD (*n* = 5/group). ^***^ *P* < 0.001 compared with the sham-operated group; ^###^ *P* < 0.001 compared with the untreated groups.

In Figure 7, the results illustrated a higher lung injury score in the TBI groups than in the sham-treated group (in which lung injury score was almost zero). These lung injury scores were significantly lower in the TM-1 treated groups (3 or 4 h after injury) than in the TBI + veh group. When the rats were sacrificed at 8 h or 24 h after TBI, the lung injury score was markedly lower in the TM-1 treated groups (3 or 4 h after injury) than in the TBI + veh groups. No significance was found between the rats administered TM-1 (10 mg/kg) at 3 or 4 h after injury and sacrificed at 8 or 24 h after TBI.

### 3.8. TM-1 Administration at 3 or 4 h after Injury Showed a Positive Correlation between the Wet/Dry Weight Ratio and the Lung Injury Score at 8 and 24 h after TBI

**Figure 8:**
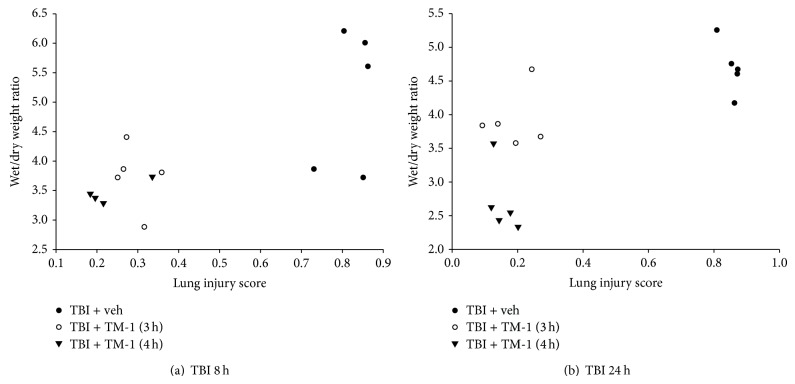
Rats administered TM-1 3 or 4 h after injury showed a positive correlation between the wet/dry weight ratio and the lung injury score. (a) The wet/dry weight ratio and the lung injury score in rats sacrificed at 8 h after TBI exhibited a positive correlation (df = 13, *r* = 0.72224) and a significant difference (*P* < 0.01) in the three groups. (b) The wet/dry weight ratio and the lung injury score in rats sacrificed at 24 h after TBI exhibited a positive correlation (df = 13, *r* = 0.72633) and a significant difference (*P* < 0.01) in the three groups.

Pearson's regression analysis showed that a positive correlation existed between the wet/dry weight ratio and the lung injury score. In Figure 8(a), lung injury score and lung wet/dry weight ratio showed a positive correlation (df = 13, *r* = 0.7224) and a significant difference (*P* < 0.05) at 3 or 4 h after injury and sacrificed at 24 h after TBI. Similarly, in Figure 8(b), and lung injury scores and lung wet/dry weight ratio were examined when animals were sacrifice at 24 h after TBI.

## 4. Discussion

Our study showed that, compared with the TBI + veh groups, a decrease in the wet/dry weight ratio was observed in the TM-1 treated groups. Histological results showed that extensive interstitial edema and infiltration of inflammatory cells occurred in injured lungs in the TBI + veh groups, but little interstitial edema and infiltration of inflammatory cells were observed in the TM-1 treated groups. These results are consistent with those of other studies [30]. The lung injury score revealed the severity of lung injury. TM-1 administered at 3 or 4 h after injury reduced the TBI-induced lung injury score measured at 8 and 24 h after TBI. To our knowledge, our study is the first to illustrate a positive correlation between the lung injury score and the wet/dry weight ratio in the same animal. Tissue edema was uniformly synchronized with tissue pathological changes. The mRNA and protein expression of AQP1 and AQP4 measured in TBI-induced lung injury was increased and attenuated by TM-1 administered (10 mg/kg) 3 or 4 h after injury, and the rats were sacrificed 8 and 24 h after TBI. The previous study demonstrated that moderate PE appeared after 8 h and peaked after 16 h of hypoxia. In addition, in lung tissue, the mRNA expression of inflammatory cytokines was upregulated after 8 h of hypoxia, and the mRNA expression of collagens was significantly increased after 8–16 h [31]. Our results are similar to those observed in the aforementioned study. Our data showed that TM-1 decreased extensive interstitial edema, infiltration of inflammatory cells, and the lung injury score. Adrenergic mechanisms may underlie this decrease. Norepinephrine and other adrenoceptor agonists are known to induce the activation of proinflammatory cytokines, such as TNF-*α*, interleukin (IL)-1, and IL-6. These cytokines are involved in the pathogenesis of PE. PE is typically accompanied by inflammation and followed by pulmonary vascular hypertrophy, permeability, and fibrosis [32]. Thaliporphine is a phenolic aporphine alkaloid obtained from Chinese herbs that possesses antioxidant and *α*-1 adrenoceptor antagonistic activity. In the previous study, thaliporphine is shown to be a partial Ca^2+^ channel agonist with strong Na^+^ and K^+^ channel-blocking activities and is attributed to the suppression of inflammatory cytokines and the production of free radicals [25]. In addition, thaliporphine is an antioxidant and *α*-1 adrenoceptor antagonist that decreased the risk of cardiac muscle necrosis [33–35]. We therefore assume that the antiadrenergic mechanisms of TM-1 may have a major role in the treatment of pulmonary injuries characterized by edema and inflammation after TBI. Chiao et al. [25] illustrated that the thaliporphine was evaluated for its antitoxic properties against toxemia induced by the bacterial endotoxin lipopolysaccharide; cardiovascular and hemodynamic changes were monitored. The authors showed that thaliporphine treatment is not associated with any significant changes in mean arterial blood pressure and heart rate or vascular hyporeactivity in response to epinephrine. We believe therefore that cardiovascular parameters in our study were unlikely affected by the thaliporphine. So we did not monitor the cardiovascular and hemodynamic changes in our study. Downregulation of AQP1 expression prevented the formation of excessive PE in chronic heart failure [17].* AQP1* knockout inhibits water transport through pulmonary capillaries and reduced high-pressure PE [16]. Furthermore, AQP1 expression increased in tuberculous pleural effusion [36]. In another study, ALI resulted in the upregulation of* AQP4* mRNA expression in rat ATII cells [21]. These results are consistent with those of our study. Our study also showed the downregulation of mRNA and protein expression of AQP1 and AQP4 as well as the positive effect of improving PE with TM-1 administration after TBI. These results may be, in part, explained by the partial Ca^2+^ channel agonist with strong Na^+^ and K^+^ channel-blocking activities of thaliporphine, which have been shown by previous studies.* AQP4* mRNA expression was upregulated in the ATII cell membrane to regulate fluid exchange between the alveolar space and the alveolar epithelium barrier and facilitated pulmonary liquid clearance in case of sodium pump transport damage during ALI [22]. Pulmonary inflammation and PE were associated with a marked reduction in* AQP1* mRNA levels in the lung [18, 19]. In addition,* AQP1* mRNA expression negatively correlated with the level of the wet/dry weight ratio [20]. In the airways,* AQP4* deletion did not impair water transport [23]. Experimental designs, therapeutic agents, animals, or cell types in the previous studies and the present study were different. However, our data imply that the mRNA and protein expression of AQP1 and AQP4 are affected by TM-1 administration and that AQPs not only regulate water balance but also regulate inflammation in lung injury after TBI. AQP5 is the main subtype in lung and expressed in type I alveolar epithelium cells [10, 37]. There is no correlation between AQP5 immunohistochemistry and alveolar septal sickness, peribronchial/vascular cuffing, perivascular edema, alveolar lipid containing macrophage, medial hyperplasia, and mast cell to perivascular edema [38]. There were some studies that demonstrated that AQP5 were not required for the physiological clearance of lung water or for accumulation of extravascular lung water in the injured lung [14, 23, 39]. Therefore, the thaliporphine derivative TM-1 could be a novel agent for reducing ALI and PE after TBI. However, further studies are required to identify the underlying mechanisms of modulation in water balance between AQPs and inflammation in the lung after TBI.

## 5. Conclusions

TM-1 simultaneously diminished the lung injury score and the wet/dry weight ratio in rats with TBI. In addition, TM-1 reduced TBI-induced PE by downregulating AQP1 and AQP4 expression. Our data suggest that the decreased expression of AQP1 and AQP4 contributes, at least in part, to the beneficial effects of TM-1 on TBI-induced PE.
